# Uterine carcinosarcoma vs endometrial serous and clear cell carcinoma: A systematic review and meta‐analysis of survival

**DOI:** 10.1002/ijgo.14033

**Published:** 2021-12-11

**Authors:** Antonio Raffone, Antonio Travaglino, Diego Raimondo, Manuela Maletta, Valentino De Vivo, Umberto Visiello, Paolo Casadio, Renato Seracchioli, Fulvio Zullo, Luigi Insabato, Antonio Mollo

**Affiliations:** ^1^ Division of Gynaecology and Human Reproduction Physiopathology Department of Medical and Surgical Sciences (DIMEC) IRCCS Azienda Ospedaliero‐Univeristaria di Bologna S. Orsola Hospital University of Bologna Bologna Italy; ^2^ Gynecology and Obstetrics Unit Department of Neurosciences, Reproductive Sciences and Dentistry University of Naples “Federico II” Naples Italy; ^3^ Pathology Unit Department of Advanced Biomedical Sciences University of Naples “Federico II” Naples Italy; ^4^ “S. Maria delle Grazie” Hospital Pozzuoli Italy; ^5^ Gynecology and Obstetrics Unit Department of Medicine, Surgery and Dentistry "Schola Medica Salernitana" University of Salerno Baronissi Italy

**Keywords:** carcinosarcoma, endometrial neoplasms, neoplasm grading, neoplasm staging, prognosis

## Abstract

**Background:**

It is unclear whether uterine carcinosarcoma (UCS) is more aggressive than endometrial serous carcinoma (SC) and clear cell carcinoma (CCC).

**Objectives:**

To compare the prognosis of UCS to that of endometrial SC and CCC, through a systematic review and meta‐analysis.

**Methods:**

Four electronic databases were searched from January 2000 to October 2020. All studies assessing hazard ratio (HR) for death in UCS *vs* SC and/or CCC. HRs for death with 95% confidence interval were extracted and pooled by using a random‐effect model. A significant *P*‐value <0.05 was adopted.

**Results:**

Six studies with 11 029 patients (4995 with UCS, 4634 with SC, 1346 with CCC and 54 with either SC or CCC) were included. UCS showed a significantly worse prognosis than SC/CCC both overall (HR = 1.51; *P *= 0.008) and at early stage (HR = 1.58; *P *< 0.001). Similar results were found for UCS vs SC (HR = 1.53; *P *< 0.001) and UCS vs CCC (HR = 1.60; *P *< 0.001).

**Conclusions:**

Compared to SC and CCC, UCS has a significantly worse prognosis, with a 1.5–1.6‐fold increased risk of death. This might justify a more aggressive treatment for UCS compared to SC and CCC. Further studies are necessary to define the prognostic impact of different molecular subgroups.

## Abstract 2

Uterine carcinosarcoma shows worse prognosis than serous and clear cell carcinoma; this could impact patient management.

## 1 INTRODUCTION

Endometrial carcinoma is the most common gynecological malignancy in developed countries.^1^, ^2^, ^3^ The Bokhman classification recognized two types of endometrial carcinoma: type I, which is estrogen‐dependent and generally has a favorable prognosis, and type II, estrogen‐independent and with a poorer prognosis. Type I carcinomas are mainly constituted by endometrioid histotype, while type II carcinomas mainly include serous carcinoma (SC) and clear cell carcinoma (CCC).^4^ Although such classification is now considered simplistic, the distinction into endometrioid and non‐endometrioid is still crucial in terms of patient management.^4^, ^5^, ^6^ In fact, SC and CCC are considered “high grade” by definition, and both the ESGO and the NCCN guidelines recommend a more aggressive treatment for these histotypes compared to G3 endometrioid carcinomas.^2^, ^5^, ^6^

In addition to SC and CCC, two further histotypes have more recently been included in the classification of endometrial carcinoma: uterine carcinosarcoma (UCS) and undifferentiated/dedifferentiated carcinoma (UDC‐DDC).^2^, ^7^, ^8^

UCS, also called malignant mixed Müllerian tumor, is a biphasic epithelial‐stromal neoplasm characterized by a carcinomatous component and a sarcomatous component.^7^ The classification of UCS has long since been debated. Grouped among the “mixed Müllerian tumors” in the former (2014) WHO classification,^9^ UCS has previously been lumped together with uterine sarcomas in terms of patient management.^10^ To date, UCS is biologically considered as an endometrial carcinoma which secondarily exhibits a mesenchymal differentiation.^2^, ^7^ Based on its aggressive behavior, UCS is now lumped together with SC and CCC for management purpose in both the ESGO and the NCCN guidelines.^5^, ^6^

UDC‐DDC is an uncommon entity which has only been recognized in the last 10 years; it shows some similarities with UCS, such as the presence of high‐grade dyscohesive cells which lacks epithelial differentiation, making the differential diagnosis difficult in some cases.^2^, ^8^ As UCS, UDC‐DDC is placed among the “high‐risk histologies” of endometrial carcinoma in the guidelines.^5^, ^6^

However, there is evidence that UCS and UDC‐DDC may be even more aggressive than SC and CCC.^11^, ^12^, ^13^ On this account, the NCCN guidelines recommend adjuvant treatment for UCS and UDC‐DDC even when limited to the endometrium with no residual tumor on the final hysterectomy specimen.^6^ By contrast, there are some studies that suggested a similar prognosis between these two histotypes and SC/CCC.^14^, ^15^, ^16^

Based on these considerations, the objective of this study was to assess whether UCS is consistently more aggressive than SC or CCC, through a systematic review and meta‐analysis. UDC‐DDC was not considered because there are too few studies assessing its prognosis in comparison to other histotypes. Our aim was to determine if a more aggressive management is justified in UCS.

## 2 MATERIALS AND METHODS

### 2.1 Study protocol

The several stages of the systematic review and meta‐analysis (electronic search, study selection, data extraction, risk of bias assessment, data analysis) were defined before the beginning of the study, based on methods of previous studies.^3^, ^17^ Two authors (AT, AR) independently performed all reviews. All authors consulted in the case of issues/disagreements. The review was reported by following the PRISMA statement.^18^

### 2.2 Electronic search and study selection

Four electronic databases (Scopus, PubMed, Web of Science, Google Scholar) were searched from January 2000 to October 2020 by using the following combination of text words: (uterine OR endometr*) AND (carcinosarcoma OR malignant mixed Mullerian) AND (serous OR clear cell). The data search was conducted in November 2020. All studies reporting hazard ratios (HR) for overall survival in UCS vs other high‐grade endometrial carcinoma were included. Exclusion criteria were: overlapping patient data, different endometrial carcinoma histotypes lumped together in the analysis, reviews. Reference list of eligible studies were also searched.

### 2.3 Data extraction

Data were extracted from primary studies according to the “PICOS”,^18^ as follows: P (population) = patients with “high‐risk histologies” of endometrial carcinoma^6^; I (intervention, risk factor) = UCS; C (comparator) = SC or CCC; O (outcome) = overall survival; S (study design) = survival cohort study. The main extracted data were HR for overall survival with 95% confidence interval (CI) for UCS versus SC, CCC or both. HRs values reported in the primary studies were used; no software was used to extract data.

### 2.4 Risk of bias assessment

The risk of bias within studies was assessed by using the QUADAS‐2^19^ as a base and adapting it to the study items, as previously described.^7^, ^8^ Four domains were assessed: (1) Patient selection (were selection criteria and period of enrollment reported? Were patients consecutive?); (2) Index test (were histological slides reviewed to confirm the histotype?); (3) Reference standard (were survival analysis adjusted for clinico‐pathological factors?); and (4) Flow and timing (was the mean follow‐up duration ≥2 years?). For each domain, the judgement was categorized as “low”, “unclear” or “high” risk of bias, as previously described.^7^, ^8^

### 2.5 Data analysis

HRs with 95% CI were pooled by using a random effect model, based on the assumption that results may vary based on factors such as geographical setting. Results were reported graphically on forest plots with 95% confidence interval (CI). Statistical heterogeneity among studies was quantified by using Higgins’ inconsistency index (*I* ^2^), as previously described.^3^, ^17^ The risk of bias across studies was assessed through a funnel plot of standard error by logHR; this allowed us to assess whether smaller and less accurate studies might have an excessive impact on the results. Data analysis was performed by using Comprehensive Meta‐Analysis (Biostat).

## 3 RESULTS

### 3.1 Study characteristics

**FIGURE 1:**
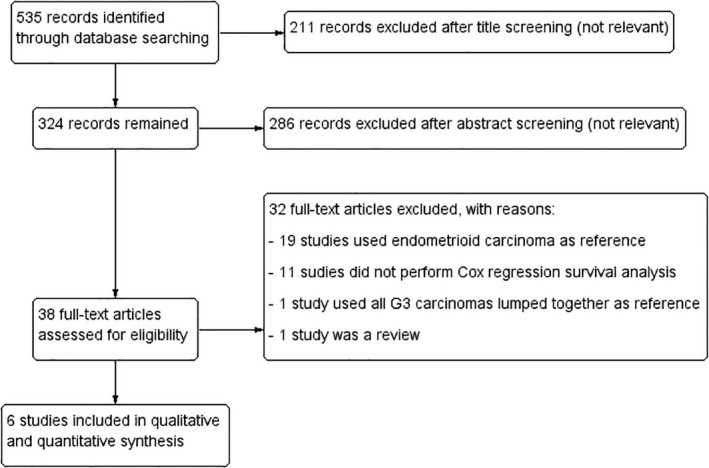
Flow diagram of studies identified in the systematic review (Prisma template [Preferred Reporting Item for Systematic Reviews and Meta‐analyses])

Six studies with 11029 patients (4995 with UCS, 4634 with SC, 1346 with CCC and 54 with either SC or CCC) were included.^14^, ^16^, ^20^, ^21^, ^22^, ^23^ The process of study selection is reported in Figure 1. Three studies selected all eligible patients independently of FIGO stage,^14^, ^16^, ^20^ while the other three studies only selected early‐stage cases (stage I^22^, ^23^ or stage I–II^21^). One study included UCS and SC,^21^ while all the remaining studies included UCS, SC and CCC. Two studies used UCS as reference of survival analysis, allowing to extract HR for both UCS vs SC and UCS vs CCC^14^, ^16^; among the other studies, two used SC as reference,^21^, ^23^ one used CCC^22^ and one used SC and CCC lumped together (Table 1).^20^

**TABLE 1:** Characteristics of the included studies

Study	Country	Database	Criterion	Period of enrollment	Sample size			Reference	Mean follow‐up (range)
					UCS	SC	CCC		
Amant 2005	Belgium	Katholieke Universiteit Leuven St. Maarten Hospital, Duffel Ziekenhuis Oost Limburg, Genk	All stages	1990–2004	33	54	54	CC + SC	28 (16–13) m
Felix 2011	USA	Magee‐Women's Hospital	All stages	1996–2008	81	147	73	UCS	Not reported
Desai 2014	USA	Memorial Sloan Kettering Cancer Center	Stage I‐II	2000–2011	112	60	0	SC	48 (3–139) m
Lakhman 2015	USA	Memorial Sloan Kettering Cancer Center	All stages	1998–2011	116	50	27	UCS	38 (1–168) m
Shinde 2018	USA	NCDB	Stage I	2004–2015	2701	1764	1246	CC	40 m
Venigalla 2018	USA	NCDB	Stage I	2010–2013	1952	4386	912	SC	Not reported

### 3.2 Risk of bias assessment

For the “patient selection” and the “reference standard” domains all studies were considered at low risk of bias since they exhaustively reported inclusion criteria and period of recruitment and performed multivariate Cox regression survival analysis.

For the “index test” domain, two studies reported that histological slides were reviewed and were considered at low risk,^20^, ^21^ while the remaining studies were considered at unclear risk.

For the “flow and timing” domain two studies were considered at unclear risk since they did not report the follow‐up duration,^14^, ^23^ while the remaining studies were considered at low risk.

**FIGURE 2:**
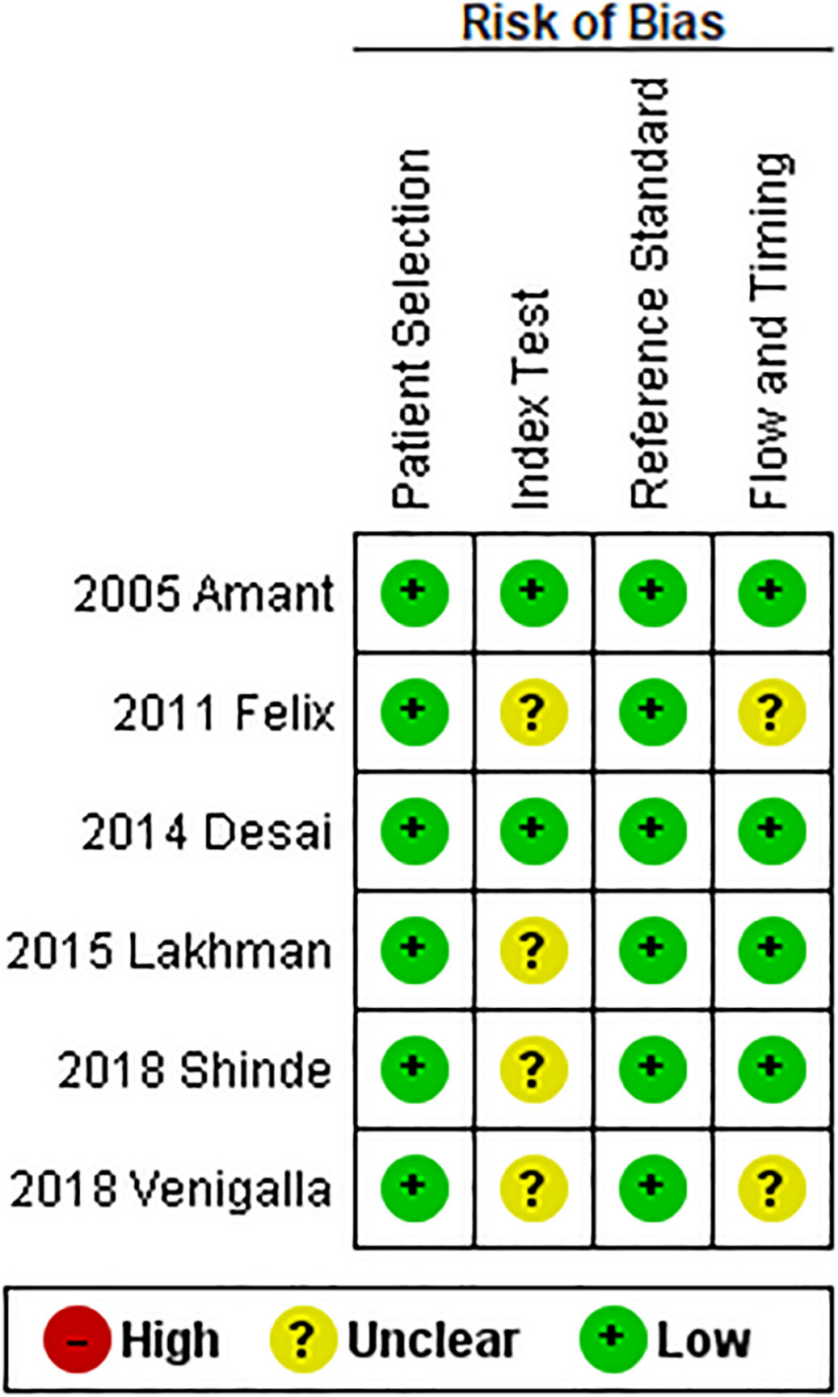
Assessment of risk of bias. Summary of risk of bias for each study; +, low risk of bias; –, high risk of bias; ?, unclear risk of bias

The results of the risk of bias assessment are reported in Figure 2.

### 3.3 Meta‐analysis

Based on the available data from the primary studies, we consulted to define the following analyses: UCS vs SC/CCC (any stage); UCS vs SC/CCC (early‐stage only); UCS vs SC; UCS vs CCC.

**FIGURE 3:**
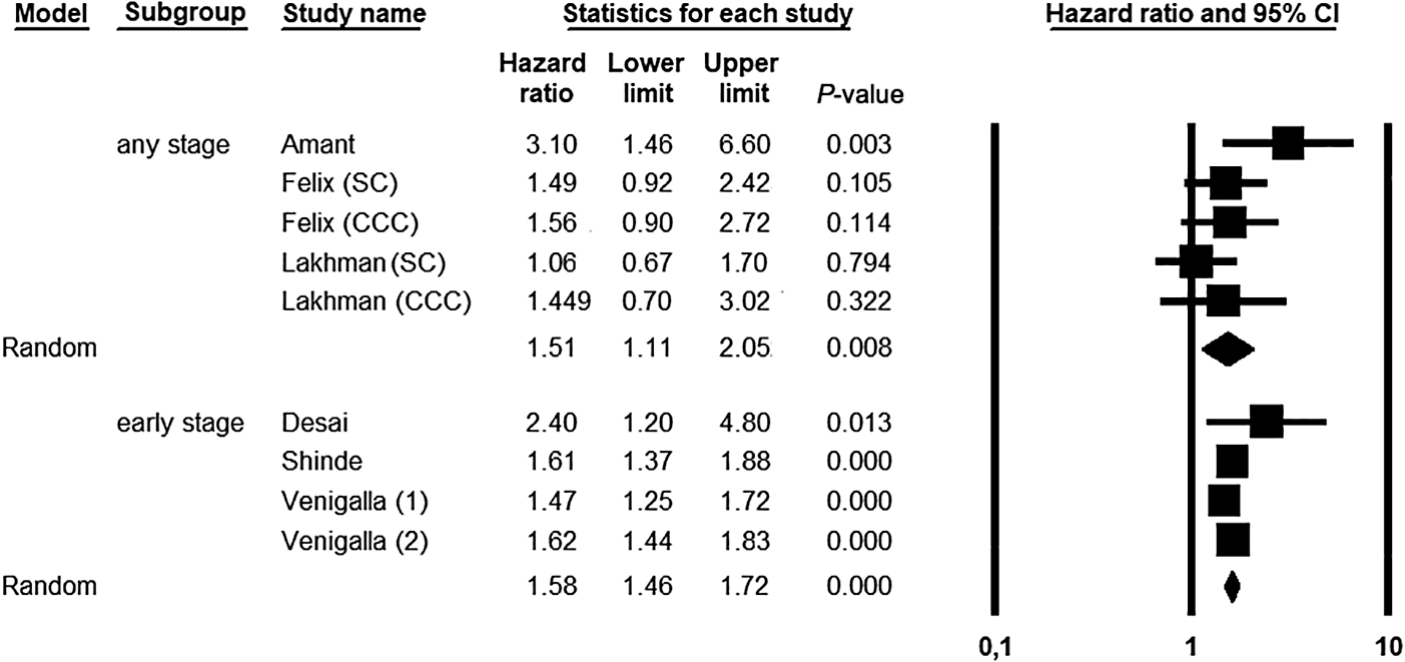
Meta‐analysis of hazard ratio for uterine carcinosarcoma (UCS) *vs* serous carcinoma (SC) and/or clear cell carcinoma (CCC) in patients at any FIGO stage (I‐IV) and early FIGO stage (I‐II). The study by Venigalla et al. included two different cohorts (1 and 2)

UCS showed a significantly increased hazard of death compared to SC/CCC independently of FIGO stage (HR = 1.51, 95% CI 1.11–2.05; *P *= 0.008); statistical heterogeneity among studies was low (*I* ^2^ = 29.09%) (Figure 3).

Considering only patients at early stage, UCS still showed a significantly increased hazard of death compared to SC/CCC (HR = 1.58, 95% CI 1.46–1.72; *P *< 0.001), with null statistical heterogeneity among studies (*I* ^2^ = 0%) (Figure 3).

**FIGURE 4:**
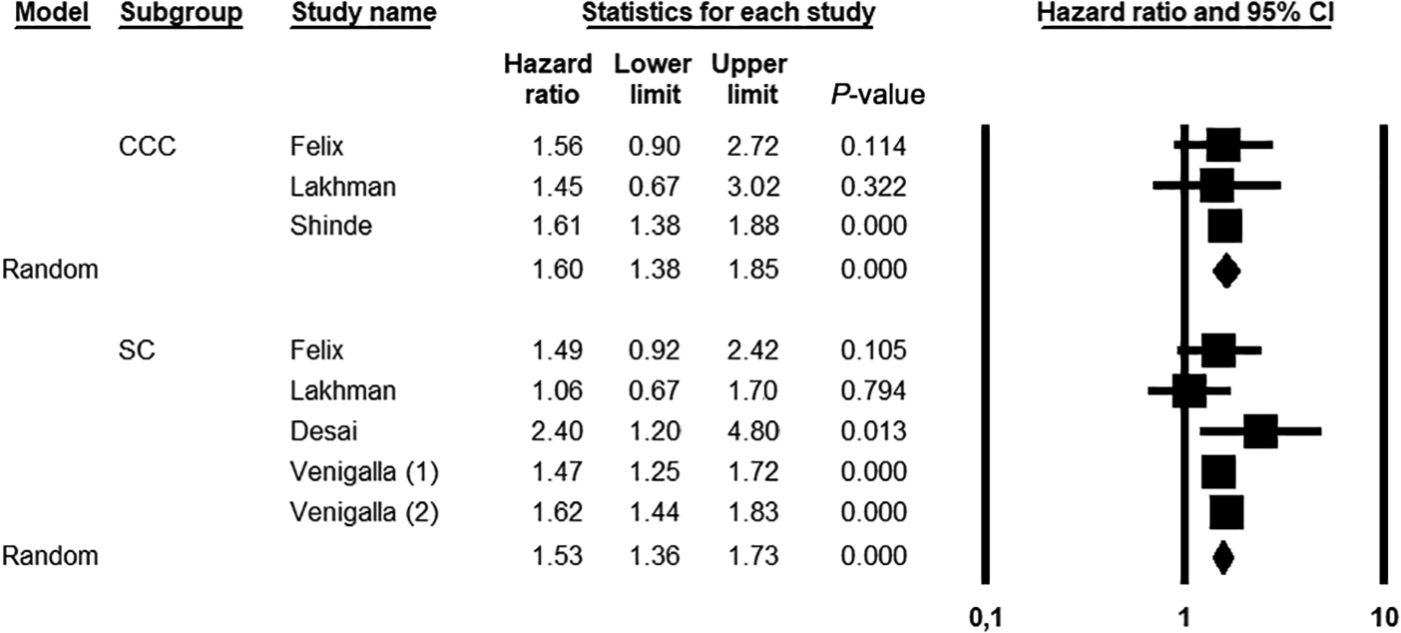
Meta‐analysis of hazard ratio for uterine carcinosarcoma (UCS) *vs* serous carcinoma (SC) and UCS *vs* clear cell carcinoma (CCC). The study by Venigalla et al. included two different cohorts (1 and 2)

Considering SC and CCC separately, UCS showed a significantly increased hazard of death compared to SC (HR = 1.53, 95% CI 1.36–1.73; *P *< 0.001), with low heterogeneity (*I* ^2^ = 20.05), and CCC (HR = 1.60, 95% CI 1.38–1.73; *P *< 0.001), with null heterogeneity (*I* ^2^ = 0%) (Figure 4).

**FIGURE 5:**
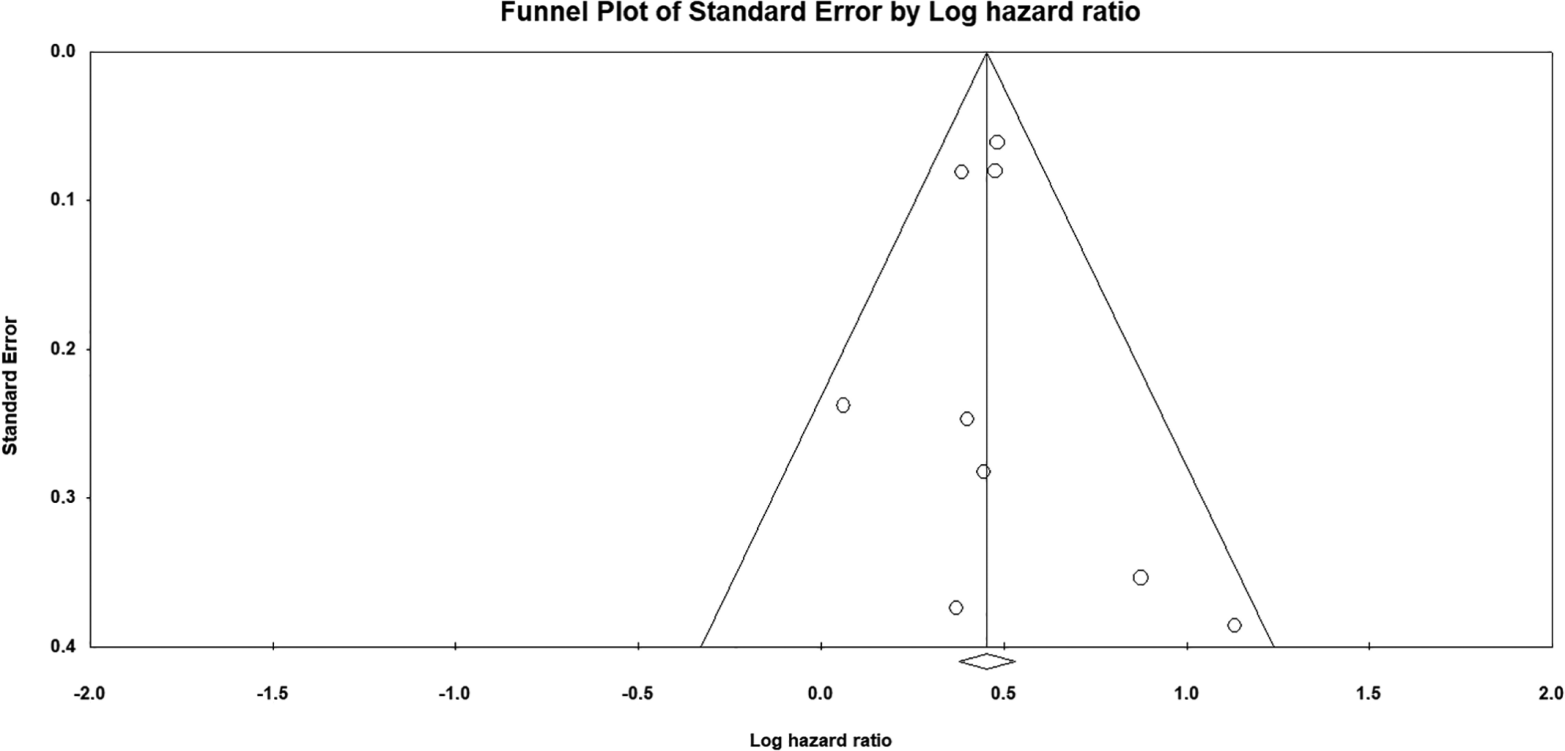
Funnel plot of standard error by log hazard ratio

The funnel plot showed no significant risk of publication bias (Figure 5).

## 4 DISCUSSION

This study showed that UCS has a significantly increased hazard of death (1.5–1.6 fold) compared to both SC and CCC, independently of FIGO stage.

Histopathological features, such as histotype, grade and lymphovascular space invasion (LVSI), are of paramount importance in the prognostic stratification of endometrial carcinoma. In fact, the current ESGO/ESTRO/ESP system identifies five risk categories to drive the patient management: low risk, intermediate risk, high‐intermediate risk, high risk, advanced/metastatic. In such system, UCS is lumped together non‐endometrioid carcinomas; this means that UCS is considered at intermediate risk when limited to the endometrium, at high‐risk in the case of FIGO stage I‐II and III‐IVA with no residual disease, and advanced/metastatic in the case of FIGO stage III‐IVA with residual disease or IVB.^5^

In recent years, The Cancer Genome Atlas (TCGA) and subsequent studies showed that endometrial carcinoma can be subdivided into four molecular prognostic subgroups, i.e. POLE‐mutated (POLEmut, good prognosis), microsatellite‐instability/mismatch‐repair deficient (MSI/MMRd; intermediate prognosis), copy‐number‐low/no specific molecular profile (CNL/NSMP; good‐to‐intermediate prognosis) and copy‐number‐high/p53‐abnormal (CNH/p53abn; poor prognosis).^3^, ^5^ Such classification has been integrated in the ESGO/ESTRO/ESP system; in such scenario, the subset of UCSs (and of other histotypes) that show a POLEmut signature is considered at low‐risk up to FIGO stage II.^5^

Despite showing an outstanding prognostic value, the TCGA might be affected by further relevant histopathological factors such as tumor budding or microcystic, elongated and fragmented (MELF) invasion pattern^24^, ^25^, ^26^; this is particularly evident for endometrioid carcinoma, which is highly heterogeneous in terms of clinico‐pathological features.^27^, ^28^, ^29^ Moreover, the prognostic significance of each molecular subgroup might be heavily affected by tumor histotype.^17^ For instance, in a large series of high‐risk endometrial carcinoma (which included endometrioid carcinomas with unfavorable prognostic factors, SC and CCC), the prognosis of the CNL/NSMP group appeared as poor as that of the CNH/p53abn group, while the POLEmut and MSI/MMRd groups showed a similar good prognosis.^30^ Instead, the MSI/MMRd group seems not to have prognostic value in UDC‐DDC.^31^ Therefore, data regarding the TCGA groups need to be integrated with histopathological prognostic factors rather than replace them. In this scenario, defining the prognostic value of highly aggressive histotypes such as UCS and UDC‐DDC is warranted.

We found that UCS showed a significantly worse prognosis than SC and CCC. Considering all patients independently of FIGO stage, UCS showed a 1.51‐fold increased hazard of death compared to SC/CCC. We also performed a subgroup analysis to compare the hazard of death in UCS to that of SC and CCC separately; we found very similar results (HR = 1.534 for SC and 1.600 for CCC). On the one hand, these results support that SC and CCC have a similar prognosis and thus should be included in the same risk category; consistently, our previous study showed that CCCs of the CNL/NSMP and CNH/p53abn groups (which represent the vast majority of CCCs^32^) had a prognosis similar to that of SC.^33^ On the other hand, UCS appears at significantly higher risk compared to the classical type II endometrial carcinomas, in agreement with our previous study.^34^ Such a result was also confirmed on the subset of patients with early‐stage disease (HR = 1.58). The latter finding is probably the most important one in terms of treatment. In fact, the management of patients with early‐stage disease may vary from follow‐up alone to several types of adjuvant treatment, including vaginal brachytherapy, external beam radiotherapy (EBRT) or systemic therapy.^5^, ^6^ Our results might support a more aggressive treatment for UCS compared to SC and CCC; this might be applied in different clinical scenarios. For tumors limited to the endometrium and completely removed at diagnostic biopsy/curettage, adjuvant treatment might be recommended for UCS but not for SC/CCC (as suggested by the NCCN guidelines^6^). For tumors limited to the endometrium and still present on the hysterectomy specimen, EBRT rather than brachytherapy might be preferable for UCS. For myoinvasive tumors limited to the uterus, the combination of EBRT and chemotherapy currently appears as the most effective approach for CNH/p53abn carcinomas (which include all SCs, about half CCCs and most UCSs)^5^; therefore, it is difficult to hypothesize a differential treatment for these tumors. A difference could be made between UCS and CCC of the CNL/NSMP group, since published data suggest that they have different aggressiveness^34^, ^35^; however, data regarding the effectiveness of chemotherapy, EBRT or both in this molecular group are scarce.^5^ Regarding the MSI/MMRd cases, a reduced sensitivity to chemotherapy has been reported, probably due to the progressive accumulation of mutations^5^, ^36^; in CCC, the MSI/MMRd signature appears associated with improved prognosis,^34^ while this is still not confirmed in UCS.^35^

All of these hypotheses need to be tested in prospective studies. It is unlikely that the aggressiveness of treatment may be modulated in advanced stages, where the prognosis is expected to be poor regardless of the histotype. In advanced carcinomas, the difference in the systemic therapy might rather be based on molecular features on the tumor. For instance, the subset of UCS which show microsatellite instability might benefit from immunotherapy.^32^

Remarkably, the NCCN guidelines propose the same approach for both UCS and UDC‐DDC, as discussed above.^6^ Since UDC‐DDC has only recently been recognized, there is much less evidence regarding its prognosis.^8^ If large series demonstrate a similar prognosis between UCS and UDC‐DDC, these two entities might be included in a separate risk category. In renal cell carcinoma, the presence of giant cells, sarcomatoid and/or rhabdoid differentiation warrants a G4 grading.^37^ We can hypothesize that such a grading might also be fit for endometrial carcinoma, with UCS and UDC‐DDC being classified as G4 carcinomas. In fact, a sarcomatoid differentiation is by definition present in UCS and may also be observed in UDC‐DDC^38^; both histotypes show evidence of epithelial‐to‐mesenchymal transition, with high‐grade dyscohesive cells that distinguish them from the other histotypes of endometrial carcinoma, and both may exhibit rhabdoid and/or giant cells.^39^, ^40^, ^41^, ^42^ Further studies are necessary in this field.

### 4.1 Strengths and limitations

To the best of our knowledge, this is the first systematic review and meta‐analysis comparing the prognosis of UCS with those of SC and CCC. We included a very large cohorts of patients, including data from national databases.^22^, ^23^ Our results were mainly based on multivariable analyses from the primary studies, which allowed us to limit the effect of confounding factors. The results were consistent when only early‐stage patients were assessed and when SC and CCC were considered separately. The low‐to‐null statistical heterogeneity found in all analyses further strengthens our findings.

A limitation of our results may lie in the fact that all but one study were from the USA; therefore, we cannot be sure that such results would be the same in other geographical areas. Another limitation may lie in the fact that most studies did not include an expert review of histological slides, as discussed in the risk of bias assessment section. In particular, the differential diagnosis between CCC and SC may sometimes be difficult due to morphologic overlap.^42^ Furthermore, UDC‐DDC has only recently been described, and older cases might have been misdiagnosed as UCS, as reported in the literature^38^; although UDC‐DDC is uncommon, we cannot exclude that it might have had an impact on the results. However, it should be noted that histological review appears not feasible in studies that assessed data from a national database.^22^, ^23^ Finally, our meta‐analysis does not take into account the molecular background of the endometrial carcinoma cases assessed. Nonetheless, SC appears a robust reference for survival analysis, since virtually all SC are *TP53*‐mutated and fall into the CNH/p53abn prognostic subgroup.^7^, ^43^, ^44^ UCS is also quite homogeneous, since it shows a CNH/p53abn signature in about 80% of cases.^7^ On the other hand, CCC appears molecularly heterogeneous, although less than endometrioid carcinoma.^33^, ^45^ Further studies in this field should involve different geographic areas, review histological slides to confirm all diagnoses and consider the molecular signature of each case.

## 5 CONCLUSION

UCS consistently showed a prognosis worse than SC and CCC, with a 1.5–1.6 times higher hazard of death. The same results were found in the subset of patients at early stage and also when SC and CCC were considered separately. This supports that, while it is appropriate to consider SC and CCC together for management purpose, UCS might need a more aggressive adjuvant treatment. The possibility of introducing a G4 grade for UCS (and possibly for UDC‐DDC) might be considered. Further studies in this field are warranted to assess the prognosis and the optimal management of these histotypes stratified according to the molecular signature.

## CONFLICT OF INTEREST

The authors report no conflict of interest.

## AUTHOR CONTRIBUTIONS

AT and AR: conception, protocol, data extraction, risk of bias assessment, data analysis, manuscript preparation, results interpretation, disagreement resolutions. DR, MM: protocol, study selection, manuscript preparation, results interpretation, disagreement resolutions. VDV and UV: protocol, electronic search, manuscript preparation, disagreement resolutions. RS: protocol, manuscript revision, results interpretation, disagreement resolutions, supervision. FZ and LI: conception, protocol, manuscript revision, results interpretation, disagreement resolutions, supervision.

## Data Availability

Data sharing is not applicable to this article as no new data were created or analyzed in this study.
